# Mapping Potential Vaccine Candidates Predicted by VaxiJen for Different Viral Pathogens between 2017–2021—A Scoping Review

**DOI:** 10.3390/vaccines10111785

**Published:** 2022-10-24

**Authors:** Zakia Salod, Ozayr Mahomed

**Affiliations:** Discipline of Public Health Medicine, University of KwaZulu-Natal, Durban 4051, South Africa

**Keywords:** vaccines, VaxiJen, viruses, reverse vaccinology, antigens, vaccinology, SARS-CoV-2, COVID-19, COVID-19 vaccines, scoping review

## Abstract

Reverse vaccinology (RV) is a promising alternative to traditional vaccinology. RV focuses on in silico methods to identify antigens or potential vaccine candidates (PVCs) from a pathogen’s proteome. Researchers use VaxiJen, the most well-known RV tool, to predict PVCs for various pathogens. The purpose of this scoping review is to provide an overview of PVCs predicted by VaxiJen for different viruses between 2017 and 2021 using Arksey and O’Malley’s framework and the Preferred Reporting Items for Systematic Reviews extension for Scoping Reviews (PRISMA-ScR) guidelines. We used the term ‘vaxijen’ to search PubMed, Scopus, Web of Science, EBSCOhost, and ProQuest One Academic. The protocol was registered at the Open Science Framework (OSF). We identified articles on this topic, charted them, and discussed the key findings. The database searches yielded 1033 articles, of which 275 were eligible. Most studies focused on severe acute respiratory syndrome coronavirus 2 (SARS-CoV-2), published between 2020 and 2021. Only a few articles (8/275; 2.9%) conducted experimental validations to confirm the predictions as vaccine candidates, with 2.2% (6/275) articles mentioning recombinant protein expression. Researchers commonly targeted parts of the SARS-CoV-2 spike (S) protein, with the frequently predicted epitopes as PVCs being major histocompatibility complex (MHC) class I T cell epitopes WTAGAAAYY, RQIAPGQTG, IAIVMVTIM, and B cell epitope IAPGQTGKIADY, among others. The findings of this review are promising for the development of novel vaccines. We recommend that vaccinologists use these findings as a guide to performing experimental validation for various viruses, with SARS-CoV-2 as a priority, because better vaccines are needed, especially to stay ahead of the emergence of new variants. If successful, these vaccines could provide broader protection than traditional vaccines.

## 1. Introduction

Vaccines have been one of the most pivotal achievements in the history of public health. The elimination of smallpox in 1980 and the near-eradication of polio have been two of the most significant achievements of immunization in the last two centuries [1,2]. Globally, vaccination saves over 386 million life years and 96 million disability-adjusted life years (DALYs) each year, preventing approximately six million deaths [3]. Hepatitis A, hepatitis B, influenza, measles, mumps, pneumococcal pneumonia, polio, rabies, rubella, coronavirus disease 2019 (COVID-19), and smallpox are among the illnesses for which vaccines are currently available [4]. However, there is a need for more efficient vaccines for these diseases. Furthermore, despite the achievements in vaccinations, many infectious diseases worldwide, such as dengue fever, hepatitis C, and herpes, are still lacking vaccines [4].

Most of the currently available vaccines were developed using a traditional vaccinology approach. Conventional vaccinology employs two methods: (i) whole pathogen vaccines (live-attenuated and inactivated), in which the relevant protective antigens are unknown; and (ii) subunit vaccines, which primarily focus on protective antigens recognized during infection [5]. However, this vaccine development strategy is (i) time-consuming, taking 5–15 years; (ii) high-risk because the pathogen must be grown in a laboratory to identify the components suitable for vaccine development; and (iii) limited to antigens expressed in vitro [6]. Reverse vaccinology (RV) can overcome these constraints, allowing for the development of more effective and innovative vaccines [6].

RV is a promising vaccine development technique focused on identifying a subset of promising antigens from pathogen proteomes through computational analysis as the first step in developing protein subunit vaccines [6]. After this first step in identifying antigens in RV, similar to conventional vaccinology, the antigens require validation in vitro and in vivo using experimental assays to confirm their protective potential. RV was first used in 2000 to identify novel antigens for developing a vaccine, Bexsero^®^, against meningococcus B [6,7]. This task was previously considered impossible by conventional vaccinology [6,7]. Bexsero^®^ received approval from the European Medicines Agency in 2013 and the United States (US) Food and Drug Administration (FDA) in 2015 [8,9]. Recently, Bexsero^®^ reduced the disease incidence by 74% in the United Kingdom and 91% in Italy [8,9]. The last two decades have seen the production of RV vaccines based on the proteome of bacterial and viral species [10]. Notably, a ribonucleic acid (RNA) vaccine against a potentially pandemic avian influenza A (H7N9) virus was created within a week in 2013 using RV that utilized the protein sequence from public databases [11]. Since the success of Bexsero^®^, many researchers have published specialized bioinformatics tools for vaccine design, known as RV prediction tools [12,13,14,15].

RV prediction tools [12,13,14,15] analyze a pathogen’s proteome to identify a group of proteins that are likely antigens as the first step toward vaccine development [6]. The predicted antigens are also known as potential vaccine candidates (PVCs). The RV approach is superior to traditional vaccinology because RV is (i) fast and efficient, taking 1–2 years; (ii) safe because the pathogen does not need to be cultured in a laboratory; and (iii) all conceivable PVCs, including those not expressed in vitro, can be identified [6]. However, one limitation of RV is that it cannot identify non-protein antigens such as polysaccharide antigens [6]. Potential antigens based on a pathogen’s protein sequences, including B and T cell epitopes in immunoinformatics [16,17,18], can be predicted using RV prediction tools. RV tools are available as standalone computer software or through online portals such as VaxiJen.

VaxiJen was the first RV website launched in 2007 [19,20,21] and is now the most widely used RV prediction tool, with the VaxiJen paper having 1480 citations in Google Scholar as of October 7, 2022. This tool predicts PVCs using an alignment-independent approach in which protein sequences are transformed into uniform equal-length vectors by auto-cross covariance (ACC). VaxiJen can predict PVCs for bacteria, viruses, tumors, parasites, and fungi. For each of these pathogen categories, five different models (with accuracies ranging between 70–89%) were created using five different datasets. The graphical interface for these five models is VaxiJen. To utilize VaxiJen, a user must first (i) enter the protein sequence(s) of a pathogen; (ii) select the appropriate pathogen type (one of the five listed above); (iii) set the desired threshold (the default is 0.5); and (iv) click the ‘submit’ button. The relevant model then runs in the background, and the output displays in VaxiJen: either ‘probable antigen’ for an antigen (PVC) or ‘probable non-antigen’ for a non-antigen (not-PVC). Any protein with an antigen probability exceeding a certain threshold qualifies as PVC. It is noteworthy that some articles citing VaxiJen for antigen prediction also reported that the resultant designed subunit vaccine protected against disease in mice [22,23,24].

From 2007 up to 2017, more than 140 researchers used VaxiJen to predict PVCs for various infectious diseases, culminating in a narrative review in 2017 [18]. However, to our knowledge, no review covered studies focused on using VaxiJen for predicting PVCs between 2017 and 2021. This review is important because the predicted PVCs can help vaccine researchers (i) design and develop a vaccine, (ii) experimentally test whether the vaccine induces protective immune responses in recipients, and (iii) identify research gaps. The objective of this study was to map the PVCs predicted by VaxiJen for various viral pathogens between 2017 and 2021.

## 2. Materials and Methods

This study is a systematic scoping review of the literature reporting on PVCs predicted by VaxiJen for different viral pathogens between 2017 and 2021. The scoping review approach was chosen because (i) it could provide a broader picture of the topic of interest (viral PVCs discovered) that may generally serve as a precursor to systematic reviews [25]; (ii) this study did not focus on a clinical question, which would be more appropriate for a systematic review, whereas the scoping review focused on mapping the evidence relating to PVCs of different viruses [25]; and (iii) it could identify research gaps [25]. We wrote a protocol for this scoping review, which was reported according to the Preferred Reporting Items for Systematic Reviews and Meta-Analyses Protocols (PRISMA-P) 2015 checklist [26,27]. We registered this protocol with the Open Science Framework (OSF) platform registries on February 17, 2022 (registration link: https://osf.io/ht8wr). A summary of the methods used in this scoping review is provided below.

**Figure 1:**
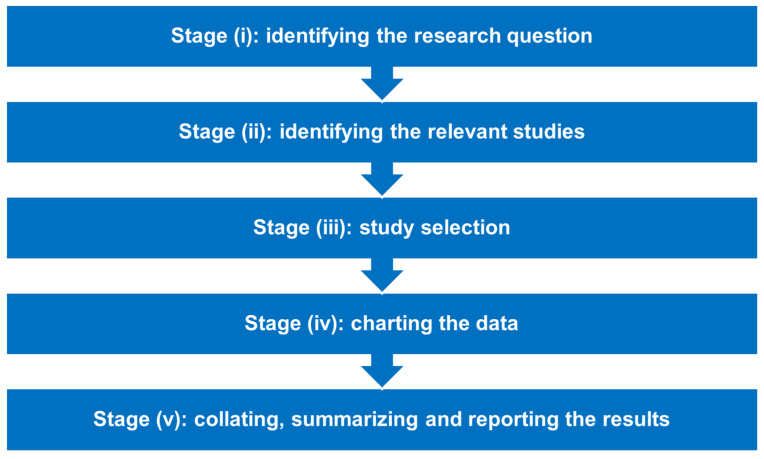
The five key stages of Arksey and O’Malley’s methodological framework for conducting a scoping review.

This scoping review employed the methodological framework of Arksey and O’Malley [28], which was later enhanced by Levac et al. [29] and the Joanna Briggs Institute (JBI) [30]. As shown in Figure 1, this framework is composed of five fundamental successive stages: (i) identifying the research question, (ii) identifying the relevant studies, (iii) study selection, (iv) charting the data, and (v) collating, summarizing and reporting the results. These stages are discussed below within the context of the present scoping review.

The abovementioned framework was used in conjunction with the Preferred Reporting Items for Systematic Reviews extension for Scoping Reviews (PRISMA-ScR) proposed by Tricco et al. [31] PRISMA-ScR provides a reporting guideline containing 20 essential items and two optional items that should be included in scoping reviews [31]. This guideline also facilitates methodological transparency and acceptance of research findings [31]. Our completed PRISMA-ScR checklist for the present scoping review is provided in Supplementary Table S2.

### 2.1. Stage (i): Identifying the Research Question

Arksey and O’Malley [28] recommended that a wide approach should be maintained when phrasing the scoping review question to increase the breadth of coverage. Therefore, the broad question for this scoping review was as follows:

What has been reported in the literature regarding potential vaccine candidates predicted by VaxiJen for different viral pathogens between 2017 and 2021?

This study utilized the population-concept-context (PCC) mnemonic, as recommended by the JBI [30], to identify the main elements of the research question (Table 1). This guidance from the PCC ensured that the study selection was in line with the aforementioned research question. The PCC mnemonic is a less restrictive substitute for the population, intervention, comparator, and outcome (PICO) mnemonic suggested for systematic reviews.

### 2.2. Stage (ii): Identifying the Relevant Studies

We conducted a search on December 23, 2021 with the search term ‘vaxijen’ in the following electronic databases: (i) PubMed [32], (ii) Scopus [33], (iii) Web of Science [34], (iv) EBSCOhost [35], and (v) ProQuest One Academic [36] (see Supplementary Table S1 for the search strategy per database). The databases listed above are both accessible and relevant to public health, allowing us to compile a comprehensive sample of the relevant literature. The eligibility criteria (inclusion and exclusion) are listed in Table 2. Initially, we planned to identify the relevant studies using a three-step approach: (i) searching the abovementioned databases, (ii) reviewing the reference lists of the included papers from the database searches to find any additional studies not found by the database searches, and (iii) hand-searching key journals to discover potentially appropriate articles that may have been missed during database and reference list searches. Notably, (i) was required, whereas (ii) and (iii) would only be undertaken if the search results from (i) were insufficient in scope and breadth. Since we found many studies in the database searches, we decided to omit the optional reference lists and journal searches.

### 2.3. Stage (iii): Study Selection

The search results from the databases were exported as a .nbib file from PubMed and as .ris files in the remaining databases. These five exported files were uploaded to Rayyan [37,38], an open-source review management software that deduplicated the articles. Rayyan supports .nbib and .ris file formats and was chosen to deduplicate articles because it has the maximum sensitivity for reference deduplication [39]. After deduplication, the remaining publications were examined in Rayyan by title and abstract (and, if necessary, by browsing the full text of an article) to identify whether the research met the inclusion requirements. The full texts of the selected articles were downloaded, screened for eligibility (Table 2), and included in this review. If we could not locate the complete text of an article online, we contacted the author(s) to obtain the full text. The screening process was guided by the main elements of this study’s research question (Table 1). ZS performed the initial screening of the articles in Rayyan, including adding reasons for exclusion in the ‘notes’ field. ZS also conducted full-text screening, and OM performed a quality assessment on 10% of the included papers.

### 2.4. Stage (iv): Charting the Data

The fourth step involved charting the data of the selected articles from stage (iii). The charting process included synthesizing and interpreting qualitative data by sifting and sorting materials using key categories and themes [28]. Arksey and O’Malley [28] suggested that the charting approach must take a broader view and that a common analytical framework should be applied to all selected studies. Therefore, a descriptive-analytical method was employed in this scoping review [28]. To this end, ZS developed a data-charting form in Microsoft Excel, which was reviewed by OM. Initially, we planned to have the following fields in the form: (i) ‘pathogen’ (name of different viruses), (ii) ‘year’ (of publication), (iii) ‘reference’, (iv) ‘key findings’ (relating to the scoping review question), and (v) ‘experimentally validated?’. However, we decided to rename ‘reference’ to ‘authors’ as it was more appropriate and clearer, include a ‘title’ field for the articles’ titles, and for studies that conducted experimental validations (‘experimentally validated?’ equals ‘Yes’), include a summary of these findings in a field called ‘experimentally validated findings’, or ‘N/A’ otherwise. ‘Experimentally validated’ referred to the verification that the vaccine-induced immune response was also directed against the native antigen. We entered the charted data into this final data-charting form and included the following fields: (i) ‘pathogen’, (ii) ‘year’, (iii) ‘authors’, (iv) ‘title’, (v) ‘key findings’, (vi) ‘experimentally validated?’, and (vii) ‘experimentally validated findings’.

### 2.5. Stage (v): Collating, Summarizing and Reporting the Results

The PRISMA flow diagram [40] was used to show the number of sources of evidence screened, evaluated for eligibility, and included in stage (iii) of the review. We employed the following three distinct stages suggested by Levac et al. [29] to present our results rigorously: (i) analyzing the data, (ii) reporting the results, and (iii) applying meaning to the results. First, based on the research objective, the research question, and Table 1 of this study, the number of papers identified by (i) year (of publication) and (ii) pathogen (the names of different viruses) was provided in a line graph and table (with fields ‘pathogen’ and ‘number of publications’), respectively. Second, to achieve the scoping review’s research question and objective, a table (data-charting form) was employed to display the results from the charted data in step (iv) in an ordered manner. Finally, the significance of the study’s findings was discussed considering research, policy, and practice (experimental validation) to aid us in formulating recommendations.

### 2.6. Ethics and Permission

This study relied solely on secondary data and did not include patient data. Therefore, ethical approval was not required for this review. Nonetheless, this study was part of a larger research project submitted for ethical consideration to the Biomedical Research Ethics Committee (BREC) of the University of KwaZulu-Natal (UKZN) in Durban, KwaZulu-Natal, South Africa. The BREC granted an exemption from ethics review for this project on March 31, 2022.

## 3. Results

### 3.1. Search Results

**Figure 2:**
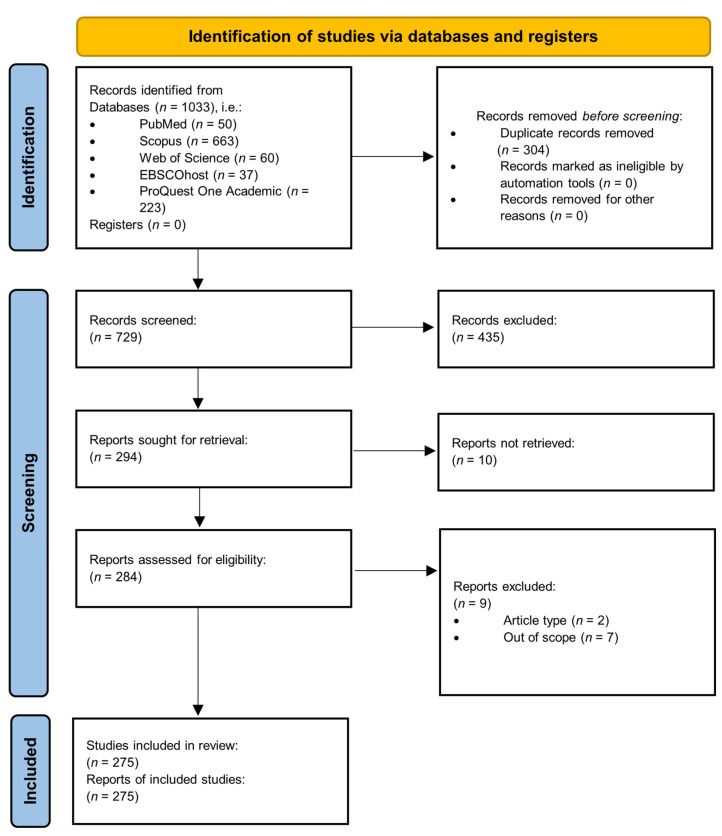
PRISMA 2020 flow diagram used for screening and selection of studies.

Database searches yielded 1033 results (50 in PubMed, 663 in Scopus, 60 in Web of Science, 37 in EBSCOhost, and 223 in ProQuest One Academic). After duplicates were removed, 729 distinct articles were screened based on their title and abstract (and, if necessary, by browsing through the full text of an article). After this screening, we attempted to retrieve 294 articles. We found the full text of 284 papers, 275 of which were eligible for inclusion (Figure 2).

### 3.2. Quantitative Overview of Articles Included in This Scoping Review

#### 3.2.1. Analysis of Publications by Year of Publication

**Figure 3:**
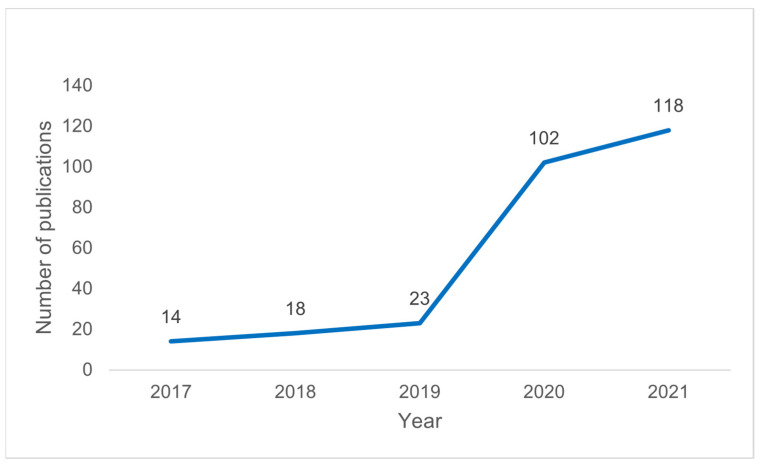
Number of publications by year.

The number of studies focusing on using VaxiJen to predict PVCs for various viruses increased exponentially between 2017 and 2021 (Figure 3). From 2017 to 2019, the number of publications gradually increased to 20% (55/275). In 2020–2021, there was an 80% increase, with a further 220 articles published. The number of studies peaked in 2021, with 118/275 papers accounting for 42.9% of the total publications.

#### 3.2.2. Analysis of Publications by Pathogen

The 275 papers included in this study were divided into 64 pathogen (virus) categories based on the article titles. Nearly half of the articles (*n* = 121; 44%) focused on severe acute respiratory syndrome coronavirus 2 (SARS-CoV-2). Human papillomavirus (HPV) (*n* = 9; 3.2%) was the second most common, followed by hepatitis C virus (*n* = 8; 2.9%) and Zika virus (ZIKV) (*n* = 8; 2.9%) tying for third place (Table 3).

### 3.3. What Has Been Reported in the Literature Regarding Potential Vaccine Candidates Predicted by VaxiJen for Different Viral Pathogens between 2017 and 2021?

As seen in the additional ‘experimentally validated?’ field included in Supplementary Table S3, only 2.9% (8/275) of the papers (‘experimentally validated?’ = ‘Yes’) conducted experimental validations of the PVCs they found predicted by VaxiJen, with 2.2% (6/275) articles mentioning recombinant protein expression [127,166,214,240,276,287]. These validations confirmed the predictions as subunit vaccine candidates, and those studies that demonstrated expression of the recombinant protein used the vectors *Escherichia coli (E. coli)* [127,166,214,240,287] and baculovirus [276]. Of the 275 articles, the following findings were the most notable for each of the top three viral pathogens. Seventy-one out of one hundred and twenty-one papers on severe acute respiratory syndrome coronavirus 2 (SARS-CoV-2) focused on the spike (S) protein. Researchers have either studied the S protein exclusively or studied the S protein along with other SARS-CoV-2 proteins to identify PVCs. Numerous antigenic (as determined by VaxiJen) T and B cell epitopes from the S protein of SARS-CoV-2 have been predicted to be PVCs. Most predicted epitopes from the S protein of SARS-CoV-2 included major histocompatibility complex (MHC) class I T cell epitopes WTAGAAAYY [89,130,136,143,147], RQIAPGQTG [43,63,92], IAIVMVTIM [43,92,146], and B cell epitope IAPGQTGKIADY [43,63,92].

Two of the 71 SARS-CoV-2 S-protein studies confirmed predictions experimentally [78,127]. The predicted T cell peptide STQDLFLPFFSNVTWFHAIHVS from the S protein of SARS-CoV-2 was antigenic in the first study, with a VaxiJen antigenicity score of 0.5545 (above the threshold of 0.4) [78]. This T cell peptide induced a robust immune response in mice with Th1-Th17 pro-inflammatory features and strong stimulation of cells involved in antibody and anti-viral cytokine production [78]. In the second study, a multivalent vaccine was developed using seven cytotoxic T cell (CTL) epitopes in the receptor-binding domain (RBD) of the S protein, three in the heptad repeat domain (HR) of the S protein, ten in the membrane (M) protein, and four epitopes in non-structural protein 13 (NSP13) of SARS-CoV-2 [127]. Additionally, the vaccine included three helper T cell (HTL) epitopes in the RBD of S protein, three in the HR of S protein, six in M, and four epitopes in NSP13 of SARS-CoV-2 [127]. VaxiJen was used to predict the antigenicity of these proteins. The vaccine candidate was safe and elicited strong antigen-specific antibody titers in mice [127].

The two major structural capsid proteins, L1 and L2, of human papillomavirus (HPV) received the most attention. Several B and T cell epitopes from HPV were discovered using predictive tools (including VaxiJen) [165,166,167,168,169,170], but they have yet to be tested experimentally. However, one of the HPV studies demonstrated in vivo that combining eight antigenic epitopes for CTL and HTL from L1 and L2 of HPV into a universal vaccine induced protective immunity in mice (~66.67% tumor-free mice; *p* < 0.05) [166].

Hepatitis C virus-based studies designed multi-epitope vaccines concentrating mainly on three viral proteins (core, NS5A, and NS5B), with antigenicity determined using VaxiJen [174,175,177,178,179,180]. One PVC included nine CTL epitopes and three HTL epitopes using the core protein of Hepatitis C virus [180]. This vaccine construct was highly antigenic, with a VaxiJen antigenicity score of 0.9882% [180]. However, studies investigating PVCs for hepatitis C virus lacked experimental validation and confirmation for their predictions.

The Zika virus (ZIKV) envelope (E) protein was the primary target of vaccine design included in five of the eight ZIKV papers [184,185,187,188,189]. One study found that the YRIMLSVHG epitope from the ZIKV E protein was the most promising for inducing a T cell immune response [184]. Another study identified ETLHGTVTV and ENSKMMLELDPPFGD as the most antigenic MHC class I and MHC class II T cell epitopes, respectively, on the ZIKV E protein [189,316]. VaxiJen confirmed that the E protein and its predicted epitopes of ZIKV were antigenic at a threshold of 0.4%. As in hepatitis C virus studies investigating PVCs, the researchers did not perform experimental validations to confirm their predictive findings in ZIKV articles.

In addition to the three studies mentioned above that experimentally validated the PVCs for the top three viral pathogens, the results of five other papers that performed experiments to confirm the predictive findings for the other viruses were as follows. A conserved epitope region (Asp348-Phe369) was discovered on the hexon capsid proteins of the fowl adenovirus of serotype 4 (FAdV-4) [287]. Asp348-Phe369 achieved an antigenicity score of 0.9293 by VaxiJen [287]. Through insertion of Asp348-Phe369 from FAdV-4 into the core protein of the hepatitis B virus, a virus-like particle (VLP) vaccine was created [287]. Compared to the commercially available vaccine (50% protection) [317], the VLP vaccine provided better protection (up to 90%) against challenge in chickens [287]. Another article reported a Crimean–Congo hemorrhagic fever (CCHF) vaccine composed of 24 epitopes (B and T cell) from the structural nucleoprotein and glycoprotein proteins of the CCHF virus [234]. These epitopes of CCHF were immunogenic (VaxiJen score above 0.4 default threshold) [234]. The novel CCHF B cell epitopes discovered in this study were validated with CCHF goat, sheep, and bovine IgG positive and negative sera, indicating that the vaccine candidate was immunogenic against CCHF [234].

An in silico study identified 23 B cell epitopes, 13 HTL epitopes, and 15 CTL epitopes in enteroviruses EV-A71, CV-A6, CV-A10, CV-A16, CV-B3 protein sequences [214]. The multivalent, multiepitope subunit vaccine constructed based on these enteroviruses epitopes was antigenic, according to VaxiJen [214]. In vitro neutralization assay experiments with three enteroviruses (EV-A71, CV-A16, CV-B3) confirmed the immunogenicity of this vaccine [214]. This experiment demonstrated that the vaccine could protect against infection with various enteroviruses [214].

Researchers predicted and confirmed the sequences GKNIGQDRDPTGVEPGDHLKERSALSYGNTLDLNSLDID and PIAGSLSGNPVNRD as linear B cell epitopes on *Seoul orthohantavirus* nucleoprotein (SHNP) [240]. BALB/c mice were immunized with recombinant protein as part of the validation [240].

Another paper predicted that the surface-exposed regions of the norovirus (NoV) GII.4 capsid protein contains five antigenic B cell epitopes (P2A-E) [276]. VaxiJen scores ranging from 0.582 to 1.358 confirmed the antigenicity of the NoV B cell epitopes [276]. These B cell epitopes were tested as synthetic peptides in wild-type mice [276]. The blocking rates in mice indicated that the predicted epitopes P2B (blocking rate: 68%), P2C (blocking rate: 55%), and P2D (blocking rate: 28%) could be used as blockade epitopes in the development of broadly reactive vaccines against NoV GII.4 [276].

## 4. Discussion

This scoping review provides an overview of the PVCs predicted by VaxiJen for various viral pathogens between 2017 and 2021. This review included 275 studies that met our inclusion criteria. Publications have increased between 2017 and 2021, with most of these 275 articles appearing between 2020 and 2021 and peaking in 2021. The research primarily focused on severe acute respiratory syndrome coronavirus 2 (SARS-CoV-2), targeting the S protein of the virus. Although VaxiJen was used to predict the antigenicity of the PVCs for various viruses in 275 publications, as with most computational studies of real-world problems, only a few papers (8/275; 2.9%) performed experimental validations, confirming the VaxiJen predictions as vaccine candidates, with 2.2% (6/275) articles mentioning recombinant protein expression.

Nearly half of the studies included in this review focused on SARS-CoV-2 infection. SARS-CoV-2 is the agent responsible for coronavirus disease 2019 (COVID-19), the worst modern-day global pandemic, first reported in China in 2019. As expected, the number of studies included in this review dramatically increased between 2020–2021, coinciding with the SARS-CoV-2-related articles published during this time. There has also been an exponential growth of various COVID-19 vaccine studies, as there are currently 172 vaccines in clinical development and 199 vaccines in pre-clinical (phases 1-4) development as of October 4, 2022 [318]. The two most well-known COVID-19 vaccines developed using the traditional vaccinology (inactivated) method are (i) COVAXIN^®^ from Bharat Biotech [319] and (ii) CoronaVac^®^ from Sinovac [320,321,322]. Some prominent COVID-19 vaccines based on other vaccine technologies and focused on the spike (S) protein include (i) vector-based vaccines from Oxford/AstraZeneca [323] and Johnson & Johnson [324] and (ii) messenger ribonucleic acid (mRNA) vaccines from Pfizer/BioNTech [325] and Moderna [318,326].

One of the main reasons for the ongoing global COVID-19 public health crisis is the emergence and spread of various SARS-CoV-2 variants caused by virus mutations [327]. Similar to some of the current COVID-19 vaccine-based studies [318,323,324,325,326,328], the SARS-CoV-2 articles included in this review primarily targeted the virus’s S protein. The former concentrated on the full-length S protein. Using the full-length S protein to create vaccines is one of the reasons for the current COVID-19 vaccine immune escape, as different viral variants circulate owing to mutations that occur primarily in this protein [329,330]. Meanwhile, the SARS-CoV-2 studies included in this review provided numerous novel results of antigenic (as defined by VaxiJen) and conserved B and T cell epitopes derived from the S protein while other studies designed vaccines based on the S protein and various other structural and non-structural SARS-CoV-2 proteins, such as M and NSP13. These findings provided valuable insights into the development of effective vaccines to combat SARS-CoV-2 and its variants, as evidenced by some articles that conducted successful experimental validations to confirm the predictions [78,127]. These SARS-CoV-2 vaccines with multiple antigenic B and T cell epitopes may be more effective than the currently licensed SARS-CoV-2 vaccines that focus on the entire S protein.

In addition to VaxiJen’s antigen predictions, the in silico studies in this review included several other investigations that have advantages for vaccine design. These vaccines were less likely to cause autoimmunity. Essentially, with RV-based vaccines, sequences in pathogen-derived antigens that are too similar to human protein sequences could selectively be avoided [331]. The vaccine constructs could cover large populations, target immune responses to specific epitopes or antigens, and be able to shape B and T cell specificities in a controlled manner. The construct could still be effective even if there are virus mutations because the vaccine candidate includes several conserved epitopes from different parts of viral protein(s). RV-based vaccines are efficient and cost-effective [262]. However, although these findings in this review are encouraging for developing novel vaccines, more in vitro studies, in vivo studies, and clinical trials are needed to confirm the predictions as subunit vaccine candidates.

This study has several implications for real-world subunit vaccine development. Vaccinologists may use the review findings to conduct experimental validations that confirm the safety and efficacy of the predictions. This review is timely given the SARS-CoV-2 vaccine-related insights discovered. The results shown in Supplementary Table S3 may be presented to vaccinologists, relevant policymakers, and funders to acquaint them with the promise of these findings for designing vaccines.

To the best of our knowledge, this is the second review to focus on VaxiJen’s PVC predictions. In 2017, VaxiJen’s authors conducted a 10-year (2007–2017) narrative review that chartered VaxiJen’s applications on bacterial, viral, parasitic, fungal, and tumor predictions [16]. On the other hand, the present scoping review focused on papers published between 2017 and 2021 to fill a gap in the literature based solely on viruses.

This review had the following limitations. First, we limited our search to English-only papers, excluding gray literature, with initial title and abstract screening completed by a single reviewer (ZS) and independent verification of extracted data completed only for a random subset of studies. However, given that we used a broad search term to search the five databases and the large number of studies included in the final review, we believe that the risk of inappropriate exclusions and significant changes to our conclusions was low. Second, although VaxiJen can predict PVCs for viruses, bacteria, fungi, parasites, and tumors [18], this review concentrated solely on viruses. These limitations resulted from the limited resources of the project.

## 5. Conclusions

This study is the first review of PVCs predicted by the VaxiJen RV tool for various viruses between 2017 and 2021. Most of the studies included in this scoping review focused on SARS-CoV-2 and were published between 2020 and 2021. Only a few papers (8/275; 2.9%) supplemented in silico PVC predictions with experimental validations to confirm the predictions as vaccine candidates, with 2.2% (6/275) articles mentioning recombinant protein expression. Given the ongoing global COVID-19 pandemic and the need for effective vaccines in the face of various viral mutations, vaccinologists may use epitope-based PVCs predictions of the SARS-CoV-2 S protein (and epitopes from S protein, together with other proteins) from the articles in this study to guide vaccine creation. In addition to carrying out experimental validations for these vaccine candidates, if successful, these vaccines may provide broader protection, target immune responses to specific epitopes or antigens of the virus, as well as several other advantages over conventional vaccines. Vaccine researchers should prioritize SARS-CoV-2 findings identified in this review because better vaccines are needed, especially to stay ahead of new variants. Researchers should also perform experimental validation for other virus studies from this review. Future research should chart VaxiJen’s applications in predicting PVCs for bacteria, fungi, parasites, and tumors, as well as viral-based articles, beginning in 2022. Future work should include non-English papers in the study if the necessary resources are available for translation, as well as gray literature.

## Figures and Tables

**Table 1:** The main elements in this study’s research question according to the JBI framework’s PCC mnemonic.

P Population	C Concept	C Context
‘different viral pathogens’	‘potential vaccine candidates predicted by VaxiJen’	‘between 2017 and 2021’

Abbreviations: JBI: Joanna Briggs Institute; PCC: population-concept-context.

**Table 2:** Criteria for inclusion and exclusion of articles in this study.

Inclusion Criteria	Exclusion Criteria
Research focused on the usage of VaxiJen for the prediction of PVCs for different viral pathogens Articles published from the year 2017–2021 Articles written in the English language Studies published in peer-reviewed journals and unpublished papers Articles that allowed access to the full text Type of studies: original articles	Research not focused on the usage of VaxiJen for the prediction of PVCs for different viral pathogens Studies published in year 2017 that were already covered in Zaharieva et al.’s [18] narrative review article Non-English articles Articles without access to the full text Non-original articles

Abbreviations: PVCs: potential vaccine candidates.

**Table 3:** Number of publications by pathogen.

Pathogen	Number of Publications	Pathogen	Number of Publications
**Severe acute respiratory syndrome coronavirus 2 (SARS-CoV-2)** [41,42,43,44,45,46,47,48,49, 50,51,52,53,54,55,56,57,58,59, 60,61,62,63,64,65,66,67,68,69, 70,71,72,73,74,75,76,77,78,79, 80,81,82,83,84,85,86,87,88,89, 90,91,92,93,94,95,96,97,98,99, 100,101,102,103,104,105,106,107,108,109, 110,111,112,113,114,115,116,117,118,119, 120,121,122,123,124,125,126,127,128,129, 130,131,132,133,134,135,136,137,138,139, 140,141,142,143,144,145,146,147,148,149, 150,151,152,153,154,155,156,157,158,159, 160,161]	121	Usutu virus (USUV) [162,163]	2
**Human papillomavirus (HPV)** [164,165,166,167,168,169,170,171,172]	9	Avian influenza A (H7N9) virus [173]	1
**Hepatitis C virus** [174,175,176,177,178,179,180,181]	8	Bovine ephemeral fever virus [182]	1
**Zika virus (ZIKV)** [183,184,185,186,187,188,189,190]	8	Canine circovirus [191]	1
Dengue virus [192,193,194,195,196,197,198]	7	Chikungunya virus and Mayaro virus [199]	1
Nipah virus (NiV) [200,201,202,203,204,205,206]	7	Dengue virus and human papillomavirus [207]	1
Ebola virus [208,209,210,211,212,213]	6	Enteroviruses [214]	1
Chikungunya virus [215,216,217,218,219]	5	Foot-and-mouth disease virus [220]	1
Human T cell lymphotropic virus type 1 (HTLV-1) [221,222,223,224,225]	5	Hepatitis and Poliovirus [226]	1
Middle East respiratory syndrome coronavirus (MERS-CoV) [227,228,229,230,231]	5	Hepatitis viruses [232]	1
Crimean-Congo haemorrhagic fever (CCHF) virus [233,234,235,236]	4	Human bocavirus 1 (HBoV1) [237]	1
Hantavirus [238,239,240,241]	4	Human herpesvirus 4 (HHV-4) or Epstein–Barr virus (EBV) [242]	1
Herpes simplex virus [243,244,245,246]	4	Human pegivirus (HPgV) [247]	1
Human coronaviruses [248,249,250,251]	4	Infectious bronchitis virus (IBV) [252]	1
Lassa virus (LASV) [253,254,255,256]	4	Influenza A virus [257]	1
Dengue virus and Zika virus [258,259,260]	3	Japanese encephalitis virus (JEV) [261]	1
Human cytomegalovirus (HCMV) [262,263,264]	3	Kaposi’s sarcoma-associated herpesvirus (KSHV) [265]	1
Human immunodeficiency virus (HIV) [266,267,268]	3	Kyasanur forest disease virus (KFDV) [269]	1
Marburg virus (MARV) [270,271,272]	3	Neural necrosis virus (NNV) [273]	1
Norovirus (NoV) [274,275,276]	3	Nipah virus (NiV) and Hendra virus (HeV) [277]	1
Rotavirus [278,279,280]	3	Oropouche virus (OROV) [281]	1
West Nile virus (WNV) [282,283,284]	3	Poliovirus [285]	1
Adenoviruses [286,287]	2	Porcine parvovirus 7 (PPV7) [288]	1
Astroviruses [289,290]	2	Porcine rubulavirus (PRV) [291]	1
Chandipura virus [292,293]	2	Rabies virus (RABV) [294]	1
Hendra virus (HeV) [295,296]	2	Saint Louis encephalitis virus (SLEV) [297]	1
Hepatitis B virus [298,299]	2	Severe acute respiratory syndrome coronavirus 1 (SARS-CoV-1 or SARS-CoV) [300]	1
Human herpes virus-5 (HHV-5) or Cytomegalovirus (CMV) [301,302]	2	Shrimp white spot syndrome virus (WSSV) [303]	1
Mayaro virus (MAYV) [304,305]	2	Sin Nombre virus (SNV) [306]	1
Newcastle disease virus (NDV) [307,308]	2	Smallpox viruses [309]	1
Respiratory syncytial virus (RSV) [310,311]	2	Tick-borne encephalitis virus (TBEV) [312]	1
Rift Valley fever virus [313,314]	2	Yellow fever virus (YFV) [315]	1

Boldface denotes the top three pathogens with the highest number of publications. The table is sorted by number of publications descending, then by pathogen ascending.

## Data Availability

Not applicable.

## Supplementary Materials

The following supporting information can be downloaded at https://www.mdpi.com/article/10.3390/vaccines10111785/s1: Table S1: Search strategy per database; Table S2: PRISMA-ScR checklist; Table S3: Included studies.
