# Supplementary material for: Mapping Potential Vaccine Candidates Predicted by VaxiJen for Different Viral Pathogens between 2017–2021—A Scoping Review
Source: Vaccines (Basel). 2022 Oct 24;10(11):1785. doi: 10.3390/vaccines10111785 (PMC9695814; doi:10.3390/vaccines10111785)
Supplement: Supplementary file 1 [file vaccines-10-01785-s001.zip › vaccines-1922649-supplementary/Supplementary Table S1 - Search strategy per database.pdf]

Supplementary File S1

Table S1: Search strategy per database

Database	Search strategy
PubMed	Search term: "vaxijen"<br>Language: English<br>Publication date: from 2017 to 2021
Scopus	ALL ("vaxijen") AND (LIMIT-TO (PUBYEAR, 2021) OR LIMIT-TO (PUBYEAR, 2020) OR LIMIT-TO (PUBYEAR, 2019) OR LIMIT-TO (PUBYEAR, 2018) OR LIMIT-TO (PUBYEAR, 2017)) AND (LIMIT-TO (DOCTYPE, "ar")) AND (LIMIT-TO (LANGUAGE, "English"))
Web of Science	(((ALL=("vaxijen")) AND PY=(2017-2021)) AND DT=(Article)) AND LA=(English)
EBSCOhost	"vaxijen" (All Fields)<br>Language: English<br>Published date: start year: 2017; end year: 2021<br>Publication type: academic journal, primary source document, working paper<br>Document type: article, journal article, research, working paper
ProQuest One Academic	"vaxijen" (Anywhere - all fields)<br>Language: English<br>Date: from 2017 to 2021<br>Document type: article, working paper/pre-print
